# Retraction: López-Valverde, N.; et al. Use of Virtual Reality for the Management of Anxiety and Pain in Dental Treatments: Systematic Review and Meta-Analysis. *J. Clin. Med.* 2020, *9*, 1025

**DOI:** 10.3390/jcm9082404

**Published:** 2020-07-28

**Authors:** Nansi López-Valverde, Jorge Muriel Fernández, Antonio López-Valverde, Luis F. Valero Juan, Juan Manuel Ramírez, Javier Flores Fraile, Julio Herrero Payo, Leticia A. Blanco Antona, Bruno Macedo de Sousa, Manuel Bravo

**Affiliations:** 1 Department of Surgery, University of Salamanca, Instituto de Investigación Biomédica de Salamanca (IBSAL), 37007 Salamanca, Spain; nlovalher@usal.es (N.L.-V.); murimuriel@gmail.com (J.M.F.); j.flores@usal.es (J.F.F.); jhpayo@usal.es (J.H.P.); lesablantona@gmail.com (L.A.B.A.); 2 Department of Biomedical and Diagnostic Sciences, University of Salamanca, Avda. Alfonso X El Sabio S/N, 37007 Salamanca, Spain; luva@usal.es; 3 Department of Morphological Sciences, University of Cordoba, Avenida Menéndez Pidal s/n, 14071 Cordoba, Spain; jmramirez@uco.es; 4 Institute for Occlusion and Orofacial Pain Faculty of Medicine, University of Coimbra, Polo I - Edifício Central Rua Larga, 3004-504 Coimbra, Portugal; brunomsousa@usal.es; 5 Department of Preventive and Community Dentistry, Facultad de Odontología, Campus de Cartuja s/n, 18071 Granada, Spain; mbravo@ugr.es

The authors of a recent published paper [1] in the *Journal of Clinical Medicine* retract their paper due to the serious flaws in the study design and data presentation. These errors were reported by two readers of the journal. The authors would like to acknowledge both readers pointing out such important mistakes. The authors apologize for not using the final data to conduct the analysis, resulting in unreliable results. The editor-in-chief and academic editors of the *Journal of Clinical Medicine* have checked this case. The editor-in-chief has approved the retraction. Both readers have been informed about the retraction decision. 

The authors apologize to readers of the *Journal of Clinical Medicine* for any inconvenience this may have caused. MDPI is a member of the Committee on Publication Ethics and takes the responsibility to uphold strict ethical policies and standards very seriously.
